# A new strategy to infer circularity applied to four new complete frog mitogenomes

**DOI:** 10.1002/ece3.3918

**Published:** 2018-03-25

**Authors:** Denis Jacob Machado, Daniel Janies, Cory Brouwer, Taran Grant

**Affiliations:** ^1^ Bioinformatics Interunits Graduate Program University of São Paulo São Paulo Brazil; ^2^ Department of Bioinformatics and Genomics UNC Charlotte Charlotte NC USA; ^3^ UNC Charlotte Bioinformatics Service Division Kannapolis NC USA; ^4^ Department of Zoology University of São Paulo São Paulo Brazil

**Keywords:** amphibians, bioinformatics, circularity, genomics, mitochondrial genome

## Abstract

We applied a novel strategy to infer sequence circularity and complete assembly of four mitochondrial genomes (mitogenomes) of the frog families Bufonidae (*Melanophryniscus moreirae*), Dendrobatidae (*Hyloxalus subpunctatus* and *Phyllobates terribilis*), and Scaphiopodidae (*Scaphiopus holbrookii*). These are the first complete mitogenomes of these four genera and Scaphiopodidae. We assembled mitogenomes from short genomic sequence reads using a baiting and iterative mapping strategy followed by a new ad hoc mapping strategy developed to test for assembly circularization. To assess the quality of the inferred circularization, we used Bowtie2 alignment scores and a new per‐position sequence coverage value (which we named “connectivity”). Permutation tests with 400 iterations per specimen and 1% or 5% chance of mutation at the ends of the putative circular sequences showed that the proposed method is highly sensitive, with a single nucleotide insertion or deletion being sufficient for circularity to be rejected. False positives comprised only 2% of all observations and possessed significantly lower alignment scores. The size, gene content, and gene arrangement of each mitogenome differed among the species but matched the expectations for their clades. We argue that basic studies on circular sequences can benefit from the results and bioinformatics procedures introduced here, especially when closely related references are lacking.

## 1 INTRODUCTION

There are currently 7,764 species of Anura (Frost, 2017), the vast majority of which have not yet had their mitochondrial genomes (mitogenomes) sequenced and studied. At the time this manuscript was written, GenBank (https://www.ncbi.nlm.nih.gov/genbank/) listed partial mitogenomes for 107 species from 71 genera and 35 families and complete mitogenomes for 238 species from 76 genera and 27 families. Increasing the diversity of studied frog mitogenomes would improve our understanding of mitogenome evolution and provide valuable information for studies ranging from phylogenetics and population genetics to genomic evolution (e.g., Bertrand et al., 2015; Mueller & Boore, 2005; Peng et al., 2017). Further, the dearth of published frog mitogenomes hampers assembly and circularity inference (i.e., validation of sequence completeness) of new mitogenomes.

To overcome the challenges in assembling anuran mitogenomes in the absence of closely related reference sequences, Machado, Lyra, and Grant (2016) optimized a strategy to efficiently reconstruct high‐quality mitogenomes directly from genomic reads using the baiting and iterative mapping approach proposed by Hahn, Bachmann, and Chevreux (2013). Machado, Lyra, and Grant validated the efficiency of this strategy as a means of assembling organelle genomes as bycatch from short genomic sequence reads sequenced using high‐throughput sequencing technology even when the total number of reads is low, and the reference belongs to distantly related taxa (i.e., different species, family, or even order). Subsequently, several authors have incorporated both the strategy and the partial mitogenomes provided by Machado, Lyra, and Grant into their methods and body of evidence (e.g., Anmarkrud & Lifjeld, 2017; Vacher, Fouquet, Holota, & Thébaud, 2016; Yuan, Xia, Zheng, & Zeng, 2016), highlighting the need to develop and improve the methods used to assemble and analyze mitogenome sequences.

A vital step of the de novo assembly of mitogenomes is the validation of sequence completeness, which, in the case of circular molecules, is assessed by testing for circularity. While assembling four novel mitogenomes of frogs from the families Bufonidae (*Melanophryniscus moreirae*), Dendrobatidae (*Hyloxalus subpunctatus* and *Phyllobates terribilis*), and Scaphiopodidae (*Scaphiopus holbrookii*) using the strategies advanced by Machado, Lyra, and Grant (which was based on partial mitogenomes only), we encountered significant difficulties in extracting the putative circular mitogenome from the final scaffolds, which included both spurious duplications of mitochondrial DNA fragments and reads that were erroneously assembled to the ends of the scaffolds.

A quick survey of the recent specialized literature (searching https://www.scopus.com and https://scholar.google.com.br for “complete mitochondrial genome” within the last 3 years) shows that many authors infer circularity through visual inspection of either reads at the ends of the assembly (e.g., Gan, Schultz, & Austin, 2014; Grau, Nuñez, Plötner, & Poustka, 2015; Vacher et al., 2016) or other more complicated methods (Cong & Grishin, 2016). However, we have observed that MITObim assemblies often produce sequences flanked by erroneous sequences that seem to have resulted from the spurious assembly of repetitive fragments. In such cases, it can be difficult or impossible to detect circularity through visual inspection—even if the entire mitogenome was assembled correctly.

Several quantitative approaches have also been proposed. A few programs, such as circules.py (distributed with MITObim), search for putative circular sequences based on *k*‐mer (i.e., substrings of length *k* contained in a sequence), overlap within an expected range of sequence length. However, these programs provide limited statistics to assess overall quality and compare alternative assemblies. More elegant solutions are available that check assembly circularity through homology searches using BLAST (Altschul, Gish, Miller, Myers, & Lipman, 1990) and comparing the size of the assembled genome to the reference (Soorni, Haak, Zaitlin, & Bombarely, 2017). Additionally, tools such as Minimus2 (Sommer, Delcher, Salzberg, & Pop, 2007) and Circlator (Hunt et al., 2015) perform circularization of genome assemblies by joining individual contigs, although they do not clip circular fragments included in larger scaffolds and focus only on identifying sequences common to both ends of a contig. All these strategies are limited to specific research questions, pipelines, sequencing technology, and availability of closely related reference genomes, creating a demand for alternatives.

Giving the abovementioned limitations and the lack of metrics to guide the choice among multiple possible options to trim the final scaffolds, we used our empirical data to develop and refine a package of computer programs validated by permutation tests to overcome the difficulty of inferring the completeness of de novo assembled mitogenomes.

## 2 MATERIALS AND METHODS

### 2.1 Whole genomic DNA sequencing

To increase the diversity of complete mitogenomes from undersampled clades, we selected four species of frogs from which to sequence mitogenomes. The mitogenome of *Scaphiopus holbrookii* (Harlan, 1835) is the first of the family Scaphiopodidae and that of the dendrobatid poison frog *Phyllobates terribilis* (Myers, Daly, & Malkin, 1978) is the first for its genus. The complete mitogenomes of the bufonid *Melanophryniscus moreirae* (Miranda‐Ribeiro, 1920) and the dendrobatid *Hyloxalus subpunctatus* (Cope, 1899) are the first of their genera, although Machado, Lyra, and Grant recently published partial mitogenomes of *M*. *simplex* (GenBank accession KT221611.1) and *H*. *yasuni* (GenBank accession KT221612.1) lacking the control region (CR).

Whole genomic DNA samples were extracted from muscle and liver samples using the DNeasy Blood & Tissue kit (Qiagen). Libraries were prepared using TruSeq Nano DNA Library Prep kit and Nextera Mate Pair (Illumina) and sequenced on Illumina HiSeq 2000/2500 machines. Macrogen Inc., Korea, performed the library preparation and DNA sequencing of *M*. *moreirae* and *S*. *holbrookii*. David H. Murdock Research Institute (DHMRI), USA, was responsible for library preparation and DNA sequencing of *H*. *subpunctatus* and *P*. *terribilis*. NCBI's GenBank, SRA, and BioSample databases contain details on mitogenomes, sequencing experiments, and specimen vouchers (see Data Accessibility).

### 2.2 Quality control

Postsequencing quality control was performed using the detailed guidelines provided by Machado et al. (2016) with some modifications. Specifically, adapter trimming for mate‐pair sequences was performed using NxTrim v0.3.0‐alpha (O'Connell et al., 2015), and all filtered reads were analyzed with FastUniq v1.1 (Xu et al., 2012) to remove putative PCR duplications. The overall quality of all sequence reads was evaluated before and after postsequencing quality control using FastQC (Andrews, 2010).

### 2.3 Mitogenome assembly

Mitogenomes were assembled using MIRA v4.0.2 (Chevreux & Suhai, 1999) and MITObim v1.8 (Hahn et al., 2013) following the baiting and iterative strategy using reference genomes from different genera or families, as discussed by Machado et al. (2016). The complete mitogenome of *Pelodytes* cf. *punctatus* II‐2011 (accession no. NC_020000.1; Pelodytidae) was used as the reference for the assembly of the *S*. *holbrookii* mitogenome. *Bufo gargarizans* (accession no. NC_020048; Bufonidae) was used as the reference for *M*. *moreirae*. Finally, *Anomaloglossus baeobatrachus* (accession no. NC_030054; Aromobatidae) was used as the reference for *H*. *subpunctatus* and *P*. *terribilis*.

We used only sequences identified as paired‐end reads after quality control for the assembly. The interleaved paired‐end sequence read file from *P. terribilis* was the largest (>150 GB disk size with ~500 M reads). Assuming mtDNA reads have a random distribution of occurrence within sequenced libraries, analyzing only a fraction of the paired‐end reads should provide adequate information to assemble the mitogenome. Therefore, we divided the reads of *P*. *terribilis* into three files of up to 52 GB disk size and ~170 M read pairs, ultimately reducing computational requirements and assembly run‐time. We validated this strategy by comparing the three scaffolds.

### 2.4 Circularity inference

**Figure 1:**
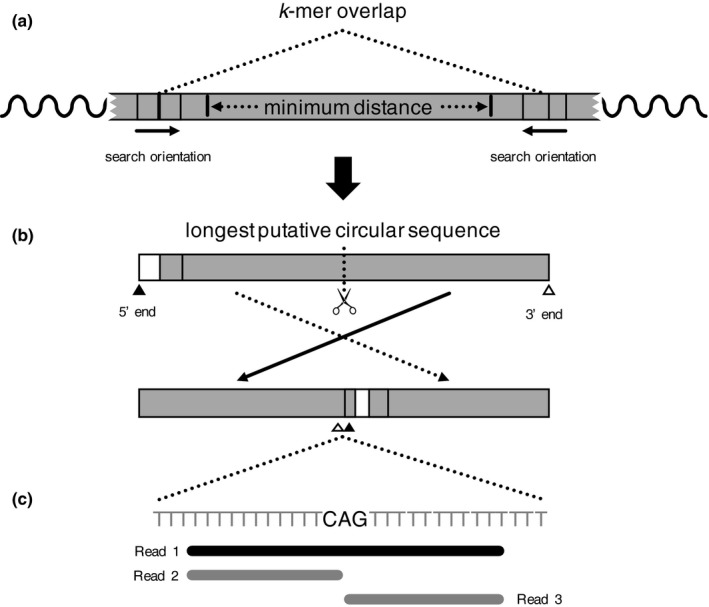
Main steps of our strategy to infer circularity. (a) We search for *k*‐mers of a specified length, from the end to the middle of the scaffold, with the condition that they are at a minimum distance from each other. (b) The longest putative circular sequence found for each *k*‐mer size is flipped, so the 5′ and 3′ ends will be adjacent to each other in the middle of the fragment. (c) Original sequence reads are remapped against the flipped putative circular sequence. All the mapped reads (represented by reads 1–3) contribute to the average alignment score. For each nucleotide, only the reads that support its position in relation to the two adjacent nucleotides (represented by read 1) are counted to determine the contiguity coverage

We divided the problem of testing for assembly circularization into two parts: The first part of the problem is to find putative overlapping sequences and use the original sequence reads to validate the circularization. To track these putative overlapping sequences, we devised a strategy that searches for identical *k*‐mers (i.e., continuous text strings) at a minimum distance from each other (Figure 1a). Once a putative mtDNA sequence is found, it is flipped and rewritten, so the ends are adjacent to each other in the middle of the sequence (Figure 1b).

In the second part of the problem, we use Bowtie2 (Langmead, Trapnell, Pop, & Salzberg, 2009) to map the original paired‐end reads to the flipped fragment. Finally, we calculate quality metrics for the assembly. These metrics include sequence similarity, coverage, and average alignment score. We also calculated a modified per‐position sequence coverage value (which we named “connectivity”) in which sequence reads that start or end at a position are excluded from the coverage calculation of that position. This allows us to quantify the number of reads that support the position of a particular nucleotide in relation to its two adjacent nucleotides (e.g., in the sequence fragment “ACT”, the connectivity of “C” ignored reads starting in or ending in “C” and considers only reads that align to the entire fragment “ACT”; Figure 1c).

Bowtie2 can align short reads quickly and efficiently, and the remaining operations can be executed in linear time, making the entire process feasible using standard personal computers. Mapping reads with Bowtie2 allowed us to test the sensitivity of our strategy to multiple *k*‐mer and mtDNA sizes.

To validate our approach, we randomly added or deleted nucleotides in 50‐bp fragments at both ends of the proposed circular sequence. We performed random deletions and additions with 1% and 5% chance, iterating the probability of modification 100 times per operation (nucleotide deletion or addition) for a total of 400 iterations per species. We then flipped sequences to make their ends adjacent to each other at the middle of the sequence. Finally, we used the flipped and modified sequences to compare the results of each iteration based on the same quality metrics described above and ranked them by the distance to the flipped sequence with no modifications (modification probability of 0%) in terms of similarity, connectivity, and average alignment scores.

### 2.5 Mitogenome annotation

We parsed the DNA in CAF format using a Python script (parseCaf.py described in Machado et al., 2016) to extract DNA data and evaluate the coverage and quality of each mtDNA element. Our preliminary de novo mitogenome annotations were performed using the mitochondrial genome annotation server MITOS (Bernt et al., 2013) with default parameters. Additional search and validation of tRNA sequences were performed using ARWEN (Laslett & Canbäck, 2008) and tRNAscan‐SE (Lowe & Eddy, 1997; Schattner, Brooks, & Lowe, 2005). Automated annotation was confirmed and edited manually by comparison with published anuran mitogenomes of closely related taxa. The CR, which typically lies between cytochrome b (*mt‐cyb*) and the LTPF tRNA cluster in neobatrachians (*mt‐tl1*,* mt‐tt*,* mt‐tp*, and *mt‐tf*) (Zhang et al., 2013), was annotated using sequence similarity searching with BLAST using default parameters (Altschul et al., 1990).

We compared our complete mitogenome sequences from *Melanophryniscus moreirae* and *Hyloxalus subpunctatus* with partial mitochondrion genome sequences available for *M*. *simplex* and *H*. *yasuni* (GenBank accession no. KT221611 and KT221612), respectively. Sequence comparisons were made using the progressiveMauve whole genome alignment algorithm (Darling, Mau, & Perna, 2010) available in Geneious version 8.1.9 (Kearse et al., 2012). Genome alignments were used as an additional verification step for the annotations and gene arrangement of these sequences, considering that gene order is not suspected to vary at this level of divergence.

### 2.6 Computational resources

Assemblies were executed on the high‐performance computing clusters ACE and Steelhead. ACE is composed of 12 quad‐socket AMD Opteron 6376 16‐core 2.3‐GHz CPU, 16 MB cache, 6.4 GT/s compute nodes (= 768 cores total), eight with 128 GB RAM DDR3 1600 MHz (16 × 8GB), two with 256 GB (16 × 16 GB), and two with 512 GB (32 × 16 GB), and QDR 4× InfiniBand (32 GB/s) networking. ACE is housed at the Museum of Zoology of the University of São Paulo (MZUSP). Steelhead comprises five high‐memory machines (Dell R815—64 AMD cores per node, 512–768 GB RAM each) and a separate computer cluster with 25 nodes, each with 16 CPUs. It is housed at the University of North Carolina at Charlotte. We performed all inferences of circularity and sequence annotation on a MacBook Pro (Retina, Mid 2012), 2.6 GHz Intel Core i7, 16 GB 1600 MHz DDR3. Parallel assembly of each mitogenome, sequentially followed by sequence annotation, was performed in 1–2 days of computer and user time.

## 3 RESULTS

### 3.1 Software

The AWA (the Tupi word for “round”) package comprises all the Python programs used for inferring circularity. Specifically, awa‐trim is used to find putative circular sequences and awa‐map is used to validate the circularization and provide basic statistics of the quality of the assembly. AWA is available at http://www.ib.usp.br/grant/anfibios/researchSoftware.html and https://gitlab.com/MachadoDJ/awa under the GNU General Public License version 3.0 (GPL‐3.0). A Wiki page with detailed user instructions and examples is available at https://gitlab.com/MachadoDJ/awa/wikis/home. The current version of AWA should be considered a beta version.

### 3.2 Inference of circularity

The four mitogenome assemblies passed the circularization tests with average alignment scores of −2.89 to −0.29 (the Bowtie2 alignment score is ≤0 in end‐to‐end mode, and the quality of the alignment is directly proportional to the alignment score). With 5% chance of adding or deleting a nucleotide at the ends of the sequence, no permutation passed the circularization test. Likewise, with 1% chance of adding a random nucleotide, no sequence was considered circular. False positives for circularity only occurred under a 1% chance of deletion and were limited to 4% of the permutations with alignment scores 1.95–14.93 times worse than the observed scores, so we expect false positives to be easy to detect. In case different putative circular sequences are obtained with different *k*‐mer sizes, we suggest using the contiguity coverage and alignment scores to choose the optimal circularization. For additional details on these experiments, see supplemental online material (Table S1).

### 3.3 Mitogenomic sequences and gene rearrangements

The mitogenomes of *H*. *subpunctatus*,* M*. *moreirae*,* P*. *terribilis*, and *S*. *holbrookii* have 16,751, 18,005, 17,702, and 16,881 bp, respectively. The final average coverage reported by MITObim is, respectively, 871.75, 196.58, 2277.35, and 1326.69X. These mitogenomes have gene contents similar to those of other vertebrates, including 13 protein‐coding genes, 22 transfer RNA (tRNA) genes, two ribosomal RNA (rRNA) genes, and one CR. Table 1 shows the base composition of each mitogenome. As in other vertebrates, the heavy strand encodes most mitochondrial genes, except for eight tRNA genes (*mt‐tp*,* mt‐tq*,* mt‐ta*,* mt‐tn*,* mt‐tc*,* mt‐ty*,* mt‐ts2*, and *mt‐te*) and *mt‐nd6* (NAD6).

**Table 1:** Number of base pairs, average sequence coverage, and nucleotide composition of the new mitogenomes

Species	Base pairs	Avg. coverage	Overall base composition (%)				
			A	C	G	T	GC
*Scaphiopus holbrookii* (Scaphiopodidae)	16,881	1,326.69	32.40	24.90	20.10	22.60	44.90
*Melanophryniscus moreirae* (Bufonidae)	18,005	196.58	30.30	24.20	14.20	31.30	38.40
*Phyllobates terribilis* (Dendrobatidae)	17,702	2,277.35	28.30	26.00	14.60	31.00	40.60
*Hyloxalus subpunctatus* (Dendrobatidae)	16,751	871.75	26.90	27.60	14.90	30.60	42.40

**Figure 2:**
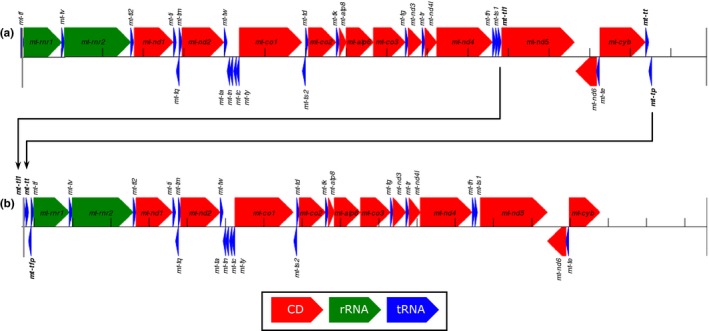
Genome arrangement in the mitochondrial genome of (a) *Scaphiopus holbrookii* and (b) *Hyloxalus subpunctatus*,* Melanoprhyniscus moreirae*, and *Phyllobates terribilis*

Gene order in the mitochondria of *H*. *subpunctuatus*,* M*. *moreirae*, and *P*. *terribilis* is identical to that of other mitogenomes of Bufonidae and Dendrobatidae. The mitogenome of *S*. *holbrookii* is the first of the family Scaphiopodidae (but a partial mitogenome of *S*. *couchii* is available; accession number JX564894) and matches the reference sequences available for the closely related families Pelobatidae (accession no. NC_008144) and Pelodytidae (accession no. NC_020000). Differences in gene order between *S*. *holbrookii* and the other three genomes are as follows: (1) the *mt‐rnr1* (12S RNA) gene is preceded by *mt‐tf* in *S*. *holbrookii* and by *mt‐tl1 *+* mt‐tt* + *mt‐tp* + *mt‐tf* in the other three mitogenomes; (2) the *mt‐nd5* (NAD5) gene is preceded by *mt‐th* + *mt‐ts1 *+* mt‐tl1* in *S*. *holbrookii* and by *mt‐th* + *mt‐ts1* only in the other genomes; (3) the CR begins immediately after the *cyb* (cytochrome *b*) gene in all but *S*. *holbrookii*, which has *mt‐cyb* and CR flanking *mt‐tt* + *mt‐tp*. See Figure 2.

Whole genome alignments between the complete mitogenome *H*. *subpunctatus* and the partial mitogenome of *H*. *yasuni* revealed identical gene order and overall similarity of 78.2%, with 12,597 identical sites. The similarity between the complete mitogenome of *M*. *moreirae* and the partial mitogenome of *M*. *simplex* was higher than the similarity between the mitogenomes of *Hyloxalus*, 82.4% with 14,017 identical sites, and the gene arrangements were also identical. Most differences between these sequences were concentrated in their CR, as expected. The CRs of our complete mitogenomes are 1,374 and 2,599 bp long for *H*. *subpunctatus* and *M*. *moreirae*, respectively. The partial CR of *H. yasuni* is 663 bp, and the partial CR of *M*. *simplex* is 515 bp long.

## 4 DISCUSSION

The four new mitogenomes presented here represent the first complete mtDNA sequences for each of the four genera, *Hyloxalus*,* Melanophryniscus*,* Phyllobates*, and *Scaphiopus*. They also represent the first complete mitogenome of Scaphiopodidae and important clades inside Bufonidae and Dendrobatidae. We expect that the increased data will help consolidate current understanding of the genome rearrangements previously proposed for these clades and corroborated here. We also anticipate that the new mitogenomes will be helpful for researchers working on mitochondrial DNA data from frogs of these three families. Moreover, these data help reduce the gaps in genome sampling of nonmodel organisms, which ultimately will collaborate to the reduction in disparities in biological understanding (Richards, 2015).

As expected based on the phylogenetic relationships and prior information on mitochondrial diversification in anurans (e.g., Irisarri et al., 2012), the mitogenome of *S*. *holbrookii* respects the vertebrate consensus mitochondrial gene order. The other mitogenomes agree with what has been proposed as a modification of gene order (*mt‐th*,* mt‐ts1*,* mt‐nd5*,* mt‐nd6*,* mt‐te*,* mt‐cyb*, CR, *mt‐tl1*, mt‐tt, mt‐tp, and *mt‐tf*) in the Neobatrachia lineage (Sumida et al., 2001; also see discussion in Xia et al., 2014). The consistency of our findings with the specialized literature is important as it suggests that the methods applied here produce reliable results.

The test of genome completeness followed a new computational pipeline with original programs called AWA. Our new approached is based on *k*‐mer matching and read mapping with Bowtie2. The use of *k*‐mer matching as a strategy to identify and align similar sequences is ubiquitous in the specialized literature on bioinformatics and computationally biology. Different functions employing *k*‐mer matching are part of the algorithms of genome aligners (e.g., MAUVE and progressiveMauve—Darling, 2004; Darling et al., 2010; ), genome assemblers (e.g., ABySS, MaSuRCA, SOAPdenovo2, and Velvet—Luo et al., 2012; Simpson, Wong, Jackman, Schein, & Jones, 2009; Zerbino & Birney, 2008; Zimin et al., 2013), tools for identification of similar sequences (e.g., BLAT and SlopMap—Kent, 2002; Zhbannikov, 2013), and multiple sequence aligners (e.g., MUSCLE and SINA—Edgar, 2004; Pruesse, Peplies, & Glöckner, 2012), among other applications. In the context of finding circular sequences via *k*‐mer matching, however, the software that is most similar to AWA is circules.py (distributed with MITObim).

The circules.py program takes a sequence in FASTA format and some parameters such as *k*‐mer size, expected sequence length, and a value of tolerance for length variation. The algorithm proceeds to find all duplicated *k*‐mers at a certain distance from each other that fits the specified range of sequence length. The circule.py program reports all putative circular sequences found this way. Depending on the particular sequence, the program may produce ambiguous results (i.e., different putative sequences supported by some duplicated *k*‐mers). In that case, the user may use the expected sequence length and number of *k*‐mer duplicates to choose among competing hypotheses. The algorithm of circules.py resembles the first part of the AWA pipeline with two relevant differences: (1) AWA takes a minimum expected sequence length instead of an anticipated sequence length with a variation threshold (i.e., AWA receives only the minimum sequence); (2) AWA reports the most extended putative circular sequence without any information on the duplicated *k*‐mers. The AWA pipeline flips the putative circular sequence and uses Bowtie2 to remap the reads to it. Instead of relying on the amount of *k*‐mer duplications, and the user rely on read coverage, read contiguity, and alignment scores to infer circularization (for details, see Section 2.4 and Figure 1).

To the best of our knowledge, the AWA pipeline is the first to introduce metrics based on the original short read data to infer sequence circularity of the assembled sequences. It also introduces an original metric, the sequence contiguity. This allowed us to infer that sequences were contiguous on the basis of both overlapping *k*‐mers on the scaffolds and high‐quality reads mapped against the putative mitogenome with an average alignment score lower than −2.9. The permutation tests provide further support to our automated approach to infer sequence circularity. These tests found only 2% false positives in all iterations. As false positives had an overall alignment score 1.95–14.93 times worse than the best scores, authors can use poor alignment scores (−3 or lower) as indications that the sequence should be reviewed and curated manually.

## 5 CONCLUSIONS

In this study, we present the first complete mitogenome of the family Scaphiopodidae and the genera *Hyloxalus*,* Melanophryniscus*, and *Phyllobates*. This increases in 1.68%, 5.26%, and 3.70% the number of anuran species, genera, and families, respectively, for which complete mitogenome sequences are known. Our approach for testing the completeness of circular DNA assemblies (presented here as a Python package named AWA) is time‐efficient and not computationally intensive. The test for mitogenome completeness can be carried out within minutes on a standard personal computer even when the file is large (i.e., 50–100 GB), and it both enables reproducibility of the tests of completeness and minimizes human error.

This procedure can also be applied to other circular genomes (e.g., chloroplasts, plasmids, covalently closed circular DNA [cccDNA] from viruses, and circular bacterial chromosomes), although applications of our strategy to nonmitochondrial sequences will be discussed in detail elsewhere.

## CONFLICT OF INTEREST

None declared.

## AUTHOR CONTRIBUTIONS

D.J.M. and T.G. conceived and designed the research. T.G. coordinated the study, provided the biological material, and performed taxonomic identifications. D.J.M. carried out bioinformatics analysis, including quality control, assembling, sequence annotation, and coding. D.J.M. also prepared tables and figures and made data and scripts publicly available. D.J. and C.B. verified and provided valuable opinions on all the bioinformatics analysis. D.J. and C.B. also contributed to useful sequencing and computational resources. All authors wrote the manuscript and gave final approval for publication together.

## DATA ACCESSIBILITY

DNA sequences: GenBank accessions KY962390–KY962393; BioProject ID PRJNA391693; BioSample accessions SAMN07271246–SAMN07271249. Homemade scripts are also available at http://www.ib.usp.br/grant/anfibios/researchSoftware.html and https://gitlab.com/MachadoDJ.
